# Supplementary material for: Functional Analysis of PdbERF109 Gene Regulation of Salt Tolerance in Populus davidiana × P. bolleana
Source: Plants (Basel). 2025 Sep 6;14(17):2800. doi: 10.3390/plants14172800 (PMC12430475; doi:10.3390/plants14172800)
Supplement: Supplementary file 1 [file plants-14-02800-s001.zip › word1.pdf]

PdbERF109 sequence:

ATGCAAAGATCTCCAAAGAGGCCCAAAATCAATGAAGCTCCGTCAGCGACTCTCTTTTCACCGC
CGGCAGCTCCACCGCTAAGATTGACCCAAGAGCAGGAGTTGGCCGTGATGGTTGCTGCTCTCA
AAAACGTAGTTTCTGGCACCGCTTCAATGGATTTCTCAAGGGAGATGAATAGTATTAATATGCCA
ATCATCACTTCACATCCACAATTTGGAAGTGCAAGCAATAACGGGAATGGTTTTTGCAACTCTAT
ATTGCCTCCATCTTCGGATCTTGACACGTGTGGTGTTTGCAAGATCAAAGGGTGCTTAGGATGCA
ACTTTTTCCCGCCAAATCAAGAAGATAAAAAGGACGACAAGAAAGGGAAACGAAAGAGAGTAA
AGAAGAATTATAGAGGTGTAAGGCAACGGCCATGGGGAAAATGGGCTGCAGAGATAAGAGAT
CCACGGAAAGCGGCAAGGGTTTGGTTAGGGACGTTTAACACTGCAGAGGAGGCGGCAAGGGC
TTATGATAAGGCAGCCATTGATTTTAGAGGGCCAAGAGCTAAGCTTAATTTTCCATTTCCTGATA
GTGGTATTGCTAGTTTTGAAGAGAGTAAAGAAAAGCAAGAAAAGCAGCAGGAAATCAGTGAGA
AGAGAAGTGAATTTGAAACGGAAATGGGGAAAGACAATGAGTTCTTGGATAATATTGTAGACG
AAGAGTTACAAGAATGGATGACGATGATGATGGATTTTGGTAATGGTGGTTCTTCCAATTCTTCC
GGTACCACAAGTGCTGCTGCTACCATTGGTTTTTAA

Protein sequence:

MQRSPKRPKINEAPSATLFSPPAAPPLRLTQEQELAVMVAALKNVVSGTASMDFSREMNSINMPIITS
HPQFGSASNNGNGFCNSILPPSSDLDTCGVCKIKGCLGCNFFPPNQEDKKDDKKGKRKRVKKNYRG
VRQRPWGKWAAEIRDPRKAARVWLGTFNTAEEAARAYDKAAIDFRGPRAKLNFPFPDSGIASFEESK
EKQEKQQEISEKRSEFETEMGKDNEFLDNIVDEELQEWMTMMMDFGNGGSSNSSGTTSAAATIGF

the minimum protein fragment：

GKDNEFLDNIVDEELQEWMTMMMDFGN
